# Eis, a novel family of arylalkylamine N-acetyltransferase (EC 2.3.1.87)

**DOI:** 10.1038/s41598-018-20802-6

**Published:** 2018-02-05

**Authors:** Qian Pan, Feng-Lan Zhao, Bang-Ce Ye

**Affiliations:** 1 Lab of Biosystems and Microanalysis, State Key Laboratory of Bioreactor Engineering, East China University of Science and Technology, Shanghai, 200237 China; 2 Collaborative Innovation Center of Yangtze River Delta Region Green Pharmaceuticals, College of Pharmaceutical Sciences, Zhejiang University of Technology, Hangzhou, 310014 Zhejiang China

## Abstract

Enhanced intracellular survival (Eis) proteins were found to enhance the intracellular survival of mycobacteria in macrophages by acetylating aminoglycoside antibiotics to confer resistance to these antibiotics and by acetylating DUSP16/MPK-7 to suppress host innate immune defenses. Eis homologs composing of two GCN5 N-acetyltransferase regions and a sterol carrier protein fold are found widely in gram-positive bacteria. In this study, we found that Eis proteins have an unprecedented ability to acetylate many arylalkylamines, are a novel type of arylalkylamine N-acetyltransferase AANAT (EC 2.3.1.87). Sequence alignment and phyletic distribution analysis confirmed Eis belongs to a new aaNAT-like cluster. Among the cluster, we studied three typical Eis proteins: Eis_*Mtb* from *Mycobacterium tuberculosis*, Eis_*Msm* from *Mycobacterium smegmatis*, and Eis_*Sen* from *Saccharopolyspora erythraea*. Eis_*Mtb* prefers to acetylate histamine and octopamine, while Eis_*Msm* uses tyramine and octopamine as substrates. Unlike them, Eis_*Sen* exihibits good catalytic efficiencies for most tested arylalkylamines. Considering arylalkylamines such as histamine plays a fundamental role in immune reactions, future work linking of AANAT activity of Eis proteins to their physiological function will broaden our understanding of gram-positive pathogen-host interactions. These findings shed insights into the molecular mechanism of Eis, and reveal potential clinical implications for many gram-positive pathogens.

## Introduction

The emergence of multidrug-resistant and extensively drug-resistant (XDR) *Mycobacterium tuberculosis* is a serious global threat. Many studies have found that the enhanced intracellular survival (Eis) protein, an acetyltransferase enzyme, is capable of conferring resistance to aminoglycoside (AG) antibiotics in *Mycobacterium tuberculosis* by acetylating multiple amines of many AGs, which are used as important second-line antituberculosis drugs^1–3^. The Eis enzyme has a unique regio-versatile AG-multiacetylating acetyltransferase activity and belongs to the Gcn5-related N-acetyltransferase (GNAT) family^2,4^. Eis homologues are found widely in mycobacteria as well as other prokaryotes. Structural and functional characterizations of several Eis enzymes that acetylate AG antibiotics, including Eis_*Mtb* (Rv2416c of *M. tuberculosis*), Eis_*Msm* (MSMEG_3513 of *Mycobacterium smegmatis*), and Eis_*Ava* (Ava_4977 of *Anabaena variabilis*), were recently performed in detail^5^. It was reported that Eis_*Msm* and Eis_*Mtb*, as secreted effector proteins, are also capable of activating DUSP16/MPK-7 (dual-protein phosphatase 16/mitogen-activated protein kinase phosphatase-7) by acetylating lysine residue Lys55, which leads to the inhibition of inflammatory responses, macrophage autophagy, and cell death, thus enhancing the survival of mycobacteria in human macrophages^6,7^.

Eis proteins are widely found in other gram-positive bacteria, in addition to mycobacteria, and they have a complex, tripartite-fold structure that is formed by two GNAT domains (IPR000182; about 140–150 amino acids) and a C-terminal animal sterol carrier protein region (SCP2 domain). The N-terminal GNAT domain is involved in acetyl-coenzyme A (Ac-CoA) binding and catalysis for acetylation. The adjoining surfaces of the N-terminal and central GNAT domains provide a large, bifurcated pocket, which is highly negatively charged for substrate AG binding^2^. This structure confers Eis the unprecedented ability to acetylate multiple amines of many AGs, some proteins, and the non-AG anti-TB drugs including isoniazid, pyrazinamide, ciprofloxacin, and capreomycin^3,6,8^. The broad specificity of Eis enzymes towards molecules containing amino residues prompted us to ask whether Eis is capable of conferring acetylation to other physiological active substrates. In this study, we sought to explore the potential of Eis to acetylate arylalkylamines.

Arylalkylamine possesses the common structural moiety Ar-C-C-N, where Ar is typically an indole or phenyl group and N represents the amino. Many arylalkylamines are biological activity compounds with different physiological functions the nervous, neuroendocrine and immune systems, including histamine, dopamine, octopamine, tyramine, tryptamine, norepinephrine, methoxytryptamine, serotonin, and 5-hydroxytryptamine^9^. The acetylation of arylalkylamines by arylalkylamine N-acetyltransferase (AANAT) is crucial for the maintenance of normal physiological functions. For example, N-acetylation of 5-hydroxytryptamine by AANAT is a rate-limiting step for the synthesis of melatonin in vertebrates. AANAT plays a unique role in vertebrate biology by controlling the rhythmic production of melatonin in the pineal gland. Hence, AANAT has been referred to as the “Timezyme”^10^. In insects, aaNATs also play important roles such as the cuticle sclerotization and the inactivation of monoamine neurotransmitters^11^.

In this study, we characterized three Eis N-acetyltransferase Eis_*Mtb*, Eis_*Msm*, and Eis_*Sen*. Interestingly, Eis proteins are all capable of acetylating arylalkylamines (histamine, octopamine, tyramine, octopamine, etc) but with different substrates specificity. The results indicated that Eis are a novel family of arylalkylamine N-acetyltransferase (EC 2.3.1.87). Eis proteins are found widely in gram-positive bacteria, including many pathogens such as *Mycobacterium*, *Enterococcus*, *Bacillus*, *Listeria*, and *Clostridium* spp. Our findings indicate that Eis may play a role in gram-positive pathogen-host interactions, which may reveal significant clinical implications.

## Results and Discussion

### Eis enzymes are found mostly in Gram-positive bacteria

Eis proteins contain N-terminal GNAT, central GNAT, and C-terminal SCP2 domains. The name “Eis” originates from the enhanced intracellular survival protein Eis of *M. tuberculosis*. The InterPro database (v48.0) comprises a total of 5152 proteins containing the Eis domain (http://www.ebi.ac.uk/interpro/entry/IPR025559). Most of these proteins (91.6%) are found in Gram-positive bacteria (2337 proteins in *Firmicutes* and 2380 proteins in *Actinobacteria*). Among 2369 *Actinomycetales* Eis proteins, 1868 proteins are found in *Mycobacteria*. As shown in Table 1, in *Firmicutes*, Eis proteins are found mostly in *Bacillales* (357), *Lactobacillales* (1460), and *Clostridiales* (434). Many pathogens, including *Mycobacterium*, *Enterococcus*, *Bacillus*, *Listeria*, and *Clostridium* spp., harbor Eis domain-containing proteins. Remarkably, most of the Eis domain (99%) is fused to another N-terminal GNAT domain to generate Eis enzymes with a unique domain organization (GNAT-Eis or GNAT-GNAT-SCP2), and exhibiting AG-acetylating acetyltransferase activity.

**Table 1:** The distribution of Eis domain in species (from InterPro database v48.0).

Species	Number of Eis proteins	
Archaea	77	
*Halobacteriaceae*		74
Bacteria	5055	
*Actinomycetales*		2369
*Bacillales*		357
*Lactobacillales*		1460
*Clostridiales*		434
Eukaryota	5	
unclassified sequences	15	

The crystal structures of seven Eis proteins have been determined, including those for Eis_*Mtb* (3R1K), Eis_*Msm* (3SXN), Eis_*Ava* (Ava_4977) (2OZG), EF_2353 (2I00) and EF_1021 (2HV2) from *Enterococcus faecalis*, Kfla_4406 (4MY3) from *Kribbella flavida*, and BAS2743 (3N7Z) from *Bacillus anthracis*. Biochemical and three-dimensional structural analyses of Eis_*Mtb*, Eis_*Msm*, and Eis_*Ava* performed to determine acetylation of AG antibiotics showed that the residues involved in CoA binding were located mainly in two GNAT domains (V85, 93RRGLLR98, 119HASE122, 126YGR128, and D260 of Eis_*Mtb*; F26, 85VAV87, 93RRGVLR98, 121SEGGIYGR128, and D258 of Eis_*Msm*; and 84FGI86, 92GDGAAI97, T121, and 252RS253 of Eis_*Ava*)^5^. The adjoining surfaces of the N-terminal and central GNAT domains provide a large, bifurcated pocket, which is highly negatively charged for substrate AG binding.

### Exploration of arylalkylamines as potential substrates of Eis

Previous studies have shown that Eis_*Mtb* is capable of deactivation the various anti-TB drugs via acetylation. Anti-TB drugs such as AGs, isoniazid, pyrazinamide, ciprofloxacin and other substrates of Eis_*Mtb* including lysine, di-lysine, tri-lysine, tetra-lysine, lisinopril, tuftsin, thymopoietin II, and peptide fragment, all possess the same part in their molecular structure, that’s the free amino group^8^. Based on this, we tried to explore whether Eis_*Mtb* could acetylate another major class of molecular containing the free amino group, arylalkylamines. To investigate whether Eis acetylates the arylalkylamines, the acetylation activities and kinetic parameters of Eis_*Mtb*, Eis_*Msm*, and Eis_*Sen* were determined for several arylalkylamines, including dopamine, tyramine, octopamine, serotonin, histamine, typtamine and phenethylamine. A typical arylalkylamine N-acetyltransferase (EC 2.3.1.87) aaNAT2 from *Aedes aegypti* was used as a control. The results about apparent kinetic constants are shown in Table 2. Eis_*Mtb* exihibits catalytic activity towards histamine and octopamine. Eis_*Msm* could not acetylate most of arylalkylamines, but exhibits affinity to octopamine and tyramine even with a high Km value. Interestingly, Eis_*Sen* showed a broad substrate specificity toward most of arylalkylamines, and exhibits the highest affinity to octopamine. The K_m_ value of Eis_*Mtb* is higher comparative to the aaNAT2 from *Aedes aegypti* but the catalytic efficiency (k_cat_/K_m_) of Eis_*Mtb* is lower, indicating Eis_*Mtb* has lower affinity and catalytic efficiency for arylalkylamines. However, not all of the identified aaNAT have good kinetic constants towards arylalkylamines. For example, an AANAT from *Saccharomyces cerevisiae* (scAANAT) was studied and its specific activity was also very low. The K_m_ values of scAANAT for serotonin was 6.5 mM and for phenylethylamine was as much as 13.3 mM (with Ac-CoA = 0.5 mM)^12^. In addition, Falcon and his co-workers characterized nonvertebrate AANAT from *C. milii*, and its K_m_ value for tyramine and serotonin were as much as 44.87 mM and 59.46 mM respectively^13^.

**Table 2:** The apparent kinetic analysis of Eis proteins and aaNAT from *Aedes aegypti*.

substrate	*K*_m_ (μM)	*k*_cat_ (s^−1^)	(*k*_cat_/*K*_m_) (M^−1^ s^−1^)
aaNAT2 from *Aedes aegypti*			
Dopamine	61 ± 1.5	10 ± 0.3	(1.7 ± 0.1) × 10^5^
Tyramine	(20 ± 4.0) × 10^2^	11 ± 0.8	(5.7 ± 1.5) × 10^3^
Octopamine	(13 ± 1.2) × 10^2^	10 ± 0.3	(8.2 ± 1.2) × 10^3^
Phenethylamine	(12 ± 1.1) × 10^2^	6.3 ± 0.2	(5.5 ± 0.7) × 10^3^
Serotonin	(8.8 ± 1.2) × 10^2^	6.3 ± 0.3	(7.3 ± 1.3) × 10^3^
Histamine	(48 ± 3.5) × 10^2^	9.2 ± 0.2	(2.0 ± 0.2) × 10^3^
Eis_*Mtb*			
Octopamine	(62 ± 19) × 10^2^	1.8 ± 0.4	(3.3 ± 1.5) × 10^2^
Histamine	(28 ± 5.2) × 10^2^	0.5 ± 0.04	(1.7 ± 0.83) × 10^2^
Eis_*Sen*			
Dopamine	(11 ± 2.4) × 10^3^	2.8 ± 0.3	(2.8 ± 1.0) × 10^2^
Tyramine	(5.7 ± 1.6) × 10^4^	18 ± 3.7	(3.7 ± 1.7) × 10^2^
Octopamine	(9.4 ± 1.6) × 10^2^	4.0 ± 0.3	(4.4 ± 1.1) × 10^3^
Phenethylamine	(1.1 ± 0.23) × 10^4^	7.2 ± 1.0	(6.7 ± 2.2) × 10^2^
Tryptamine	(4.3 ± 0.75) × 10^3^	3.0 ± 0.3	(7.3 ± 2.0) × 10^2^
Serotonin	(1.3 ± 0.18) × 10^4^	1.5 ± 0.2	(1.2 ± 0.33) × 10^2^
Eis_*Msm*			
Octopamine	(3.0 ± 2.5) × 10^4^	3.1 ± 1.1	(4.3 ± 4.0) × 10^2^
Tyramine	(1.6 ± 0.5) × 10^4^	0.48 ± 0.05	33 ± 16

The acetylation activities were measured as described in Materials and methods. Data were shown by means ± SD.

To further examine substrate specificities of Eis proteins, various acyl-CoA substrates with increasing acyl chain lengths were investigated at saturating amine substrate with the highest affinity (Table 3). The results showed that aaNAT2 revealed the broad specificities of the acyl-CoA substrates. Eis can only exhibits activity to short chain acyl-CoA substrates, acetyl-CoA and propionyl-CoA. No activities for butyryl-CoA and octanoyl-CoA was observed. A structural difference between the active sites of Eis and aaNAT2 might contribute to their substrate specificities that Eis enzymes are inactive for acyl-CoA substrates with the longer acyl chain lengths (>C3). We found a similar trend between aaNAT2 and Eis was that the K_m_ values and the relative (k_cat_/K_m_) values did decrease when increasing the acyl chain length of acyl-CoA substrates (Table 3). The decrease of k_cat_ values significantly resulted in the decline of the second-order rate. This phenomenon was also observed in aaNATL7^14,15^. The long-chain acyl-CoA substrates might perturb or block the amine substrate binding because of their extension into the binding pocket of amine substrate in Eis enzymes or aaNATL7.

**Table 3:** The apparent kinetic analysis of aaNAT and Eis.

Amine substrate	Acyl-CoA	*K*_m_ (μM)	*k*_cat_ (s^−1^)	(*k*_cat_/*K*_m_) (M^−1^ s^−1^)
aaNAT2				
Dopamine	acetyl-CoA	(6.8 ± 1.1) × 10^2^	64 ± 8.0	(9.9 ± 2.7) × 10^4^
	propionyl-CoA	(4.0 ± 1.1) × 10^2^	3.2 ± 0.40	(8.9 ± 3.5) × 10^3^
	butyryl-CoA	(2.2 ± 0.52) × 10^2^	1.4 ± 0.12	(6.8 ± 2.2) × 10^3^
	octanoyl-CoA	70 ± 13	0.16 ± 0.01	(2.4 ± 0.6) × 10^3^
Eis_*Mtb*				
Histamine	acetyl-CoA	(1.2 ± 0.27) × 10^2^	5.6 ± 0.2	(5.2 ± 1.3) × 10^4^
	propionyl-CoA	47 ± 10	0.6 ± 0.1	(1.4 ± 0.4) × 10^4^
Eis_*Sen*				
Octopamine	acetyl-CoA	(4.8 ± 1.4) × 10^2^	38 ± 5.7	(9.1 ± 3.9) × 10^4^
	propionyl-CoA	66 ± 1	2.3 ± 0.2	(3.9 ± 1.4) × 10^4^
Eis_*Msm*				
Tyramine	acetyl-CoA	(2.7 ± 0.43) × 10^2^	29 ± 1.8	(1.1 ± 0.2) × 10^5^
	propionyl-CoA	98 ± 23	2.7 ± 0.2	(2.9 ± 0.8) × 10^4^

The activities were measured as described in the Materials and methods section.

**Figure 1:**
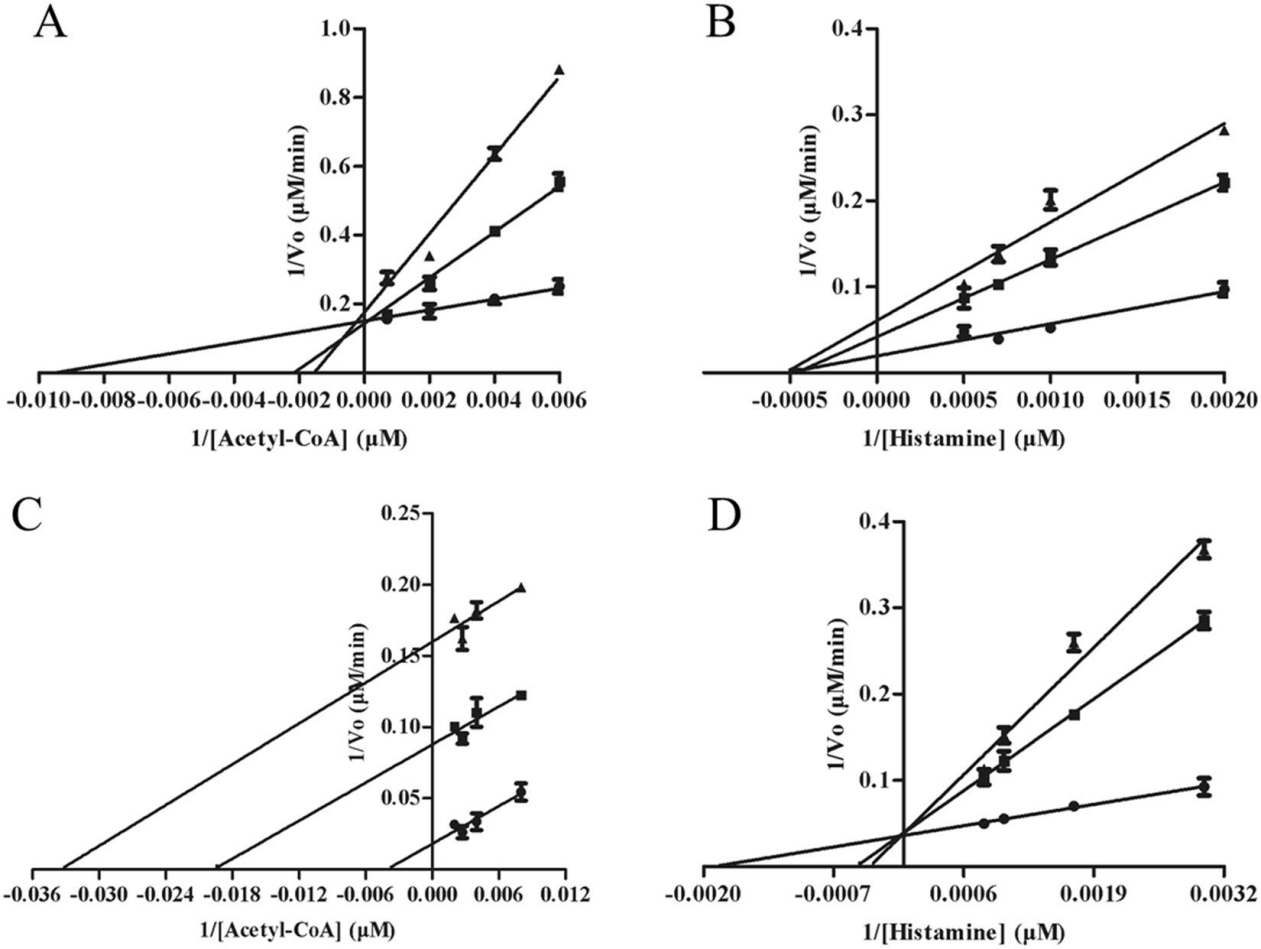
Dead-end inhibition analysis of Eis proteins. (**A**) Velocities measured at a fixed concentration of histamine (1000 μM), varying the concentration of acetyl-CoA, and varying the concentration of the inhibitor, oleoyl-CoA: 0 nM (●), 600 nM (■), and 1200 nM (▲) (Ki = 267.9 ± 68.4 nM). (**B**) Velocities were measured at a fixed concentration of acetyl-CoA (250 μM), varying the concentration of histamine, and varying the concentration of the inhibitor, oleoyl-CoA: 0 nM (●), 600 nM (■), and 1200 nM (▲) (Ki = 508.5 ± 93.8 nM). (**C**) Velocities measured at a fixed concentration of histamine (1000 μM), varying the concentration of acetyl-CoA, and varying the concentration of the inhibitor, tyrosol: 0 μM (●), 500 μM (■), and 1 mM (▲) (Ki = 130.1 ± 18.3 μM). (**D**) Velocities measured at a fixed concentration of acetyl-CoA (250 μM), varying the concentration of histamine, and varying the concentration of the inhibitor, tyrosol: 0 μM (●), 500 μM (■), and 1 mM (▲) (Ki = 175.3 ± 48.7 μM). Data shown in all experiments represent the means and standard errors (error bars) of duplicate determinations for assays repeated three times.

Arylalkylamine N-acetyltransferases usually belong to bisubstrate enzymes. The substrate binding order of multi-substrate enzymes is important to understand their catalytic mechanism. The previous researches demonstrated that *D. melanogaster* AANATA and sheep serotonin N-acetyltransferase both revealed an ordered sequential kinetic mechanism^15,16^. To scrutinize and compare the catalytic mechanism of Eis and aaNAT enzymes, we investigated the order of acetyl-CoA and arylalkylamine to bind to Eis using the dead-end inhibition experiments. Oleoyl-CoA (an analogue of acetyl-CoA) and tyrosol (a structural analogue of the amine substrate) were used as the inhibitors^14,15^. We found that oleoyl-CoA was competitive versus acetyl-CoA and noncompetitive versus histamine, with inhibition constants of 268 ± 68 nM and 509 ± 94 nM, respectively (Fig. 1). Tyrosol was uncompetitive versus acetyl-CoA and competitive versus histamine, with inhibition constants of 130 ± 18 μM and 175 ± 49 μM, respectively. These observations indicated that Eis enzymes showed an ordered sequential mechanism, similar to the catalytic mechanism of aaNAT^15,16^. N-acetylhistamine was produced with acetyl-CoA binding first followed by histamine to form a precatalytic ternary eis·acetyl-CoA·histamine complex. It is worth noting that the inhibition results (Fig. 1) are consistent with an ordered kinetic mechanism, but that other kinetic mechanisms are also possible.

### Evolution analysis of Eis enzymes and other studied aaNATs

**Figure 2:**
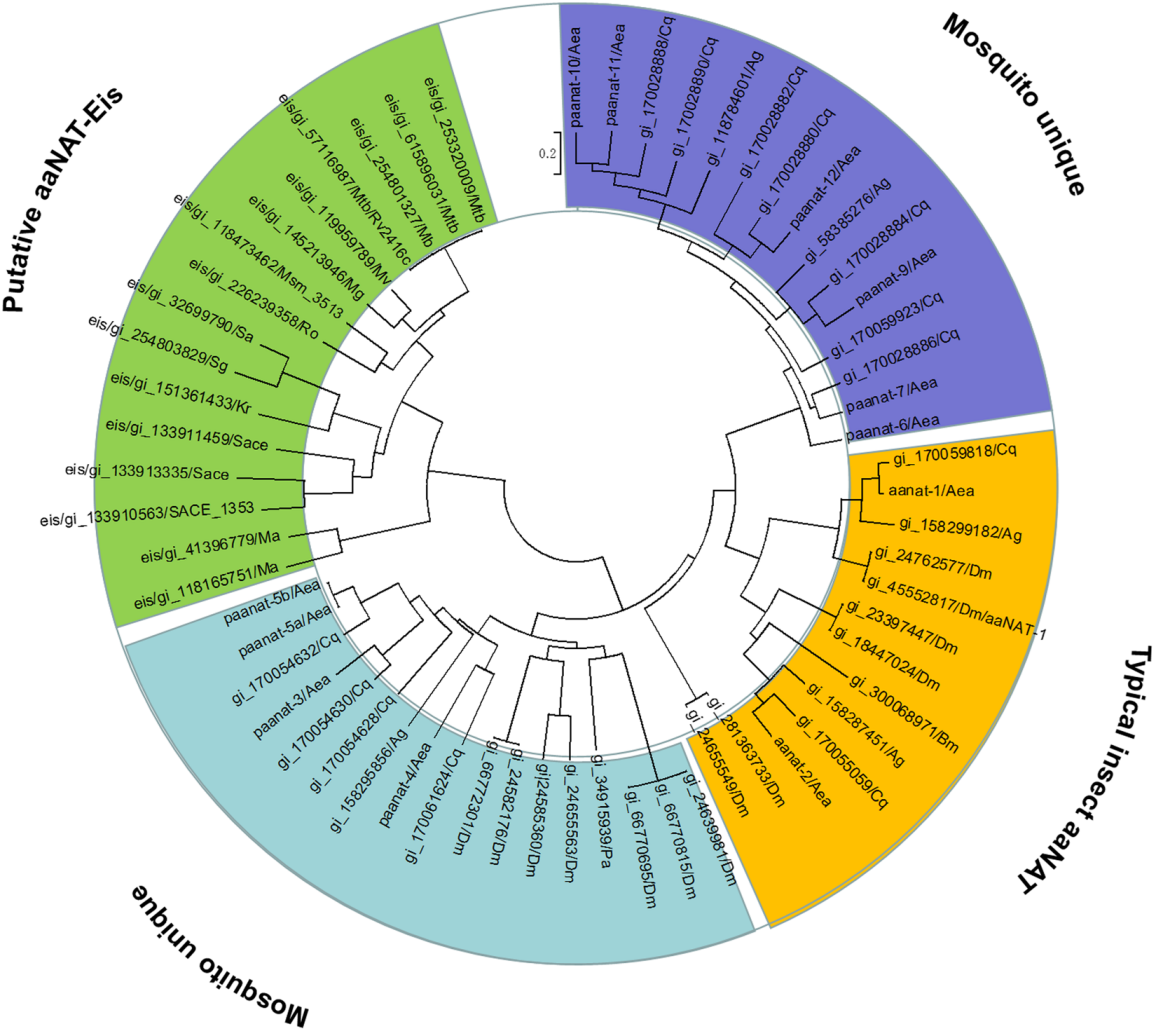
A phylogenetic tree of identified and putative aaNATs from mosquitoes, insects and bacteria. Blue and cyan areas covered two mosquito unique clusters; orange area covered typical insect aaNAT cluster; Green area covered putative aaNAT-Eis cluster. *Aea, Aedes aegypti; Ag, Anopheles gambiae; Bm, Bombyx mori; Cq, Culex quinquefasciatus; Dm, Drosophila melanogaster; Pa, Periplaneta americana; Mtb, Mycobacterium tuberculosis; Msm, Mycobacterium smegmati; Sace, Saccharopolyspora erythraea; Kr, Kineococcus radiotolerans; Ma, Mycobacterium avium*.

To understand the phylogenetic relationship between Eis and other known aaNATs, we constructed a phylogenetic tree (Fig. 2). In this study, most of aaNATs chosen on the phylogenetic tree are based on previous reports^17^. The sequences of GNAT domains in Eis enzymes were used for phylogenetic analysis. Phyletic distribution analysis confirmed four major clusters, termed clusters 1 (orange), 2 (cyan), 3 (blue), and 4 (green). Cluster 1 represent typical insect aaNATs, all identified insect aaNATs are located in cluster 1. The aaNAT-2 in cluster 1 was selected as a control. Mosquito aaNATs were clustered into two areas (cyan and blue). We found that all Eis homologues could be clustered into the one clade, including the Eis_*Mtb*, Eis_*Msm*, and Eis_*Sen* proteins from this set.

### N-acyltransferase superfamily sequence alignment and structural comparison of aaNAT2 from *Ae. aegypti* and the Eis enzymes

**Figure 3:**
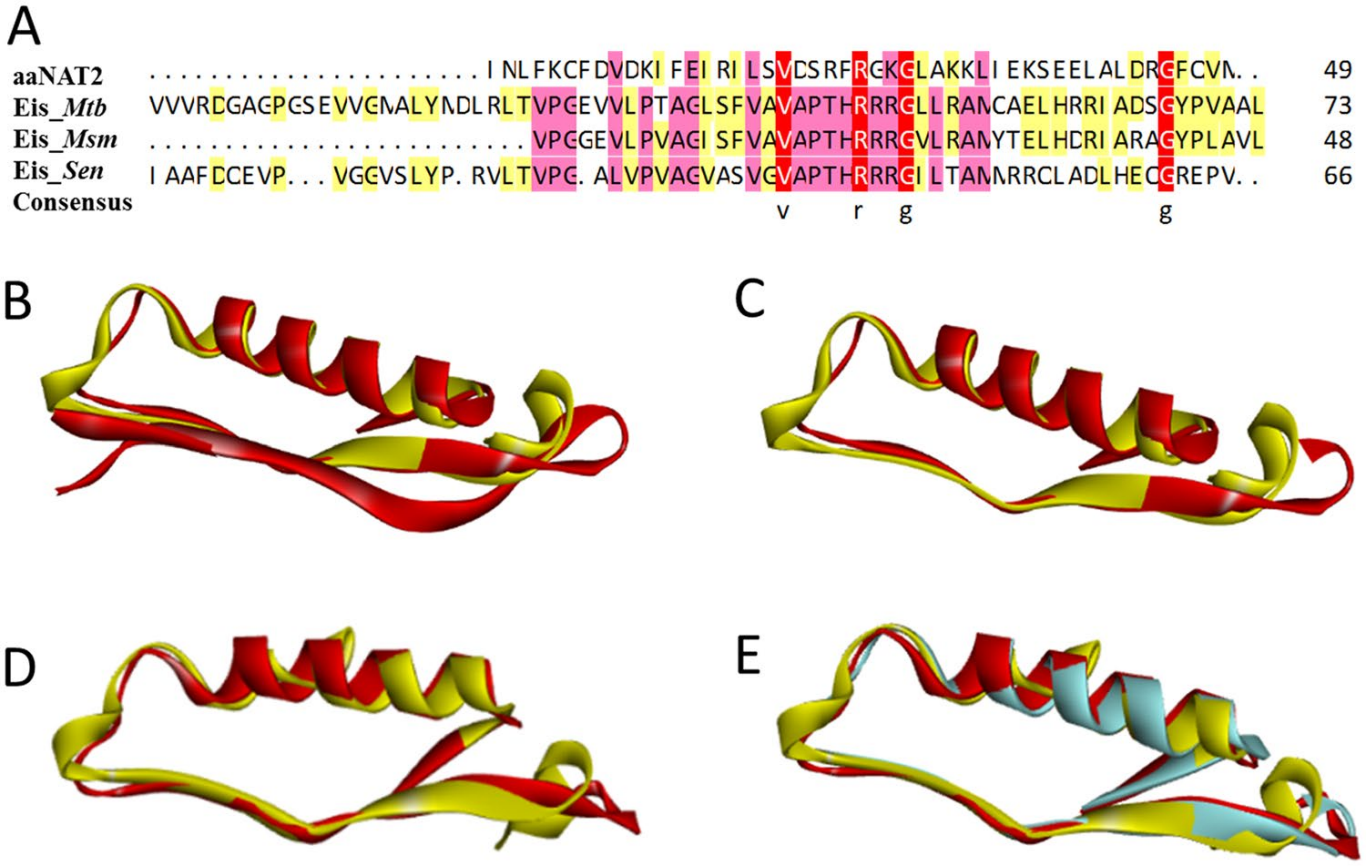
Sequence alignment and structural comparison of aaNATs from *Ae. aegypti* and Eis proteins. (**A**) Multiple sequence alignment of N-acyltransferase superfamily region of Eis_*Mtb*, Eis_*Msm*, and Eis_*Sen* with the region of aaNAT2 from *Ae. aegypti*. (**B**) Superimposition of the N-acyltransferase superfamily structure within Eis_*Mtb* (colored in red) onto the structure within aaNAT2 (colored in yellow). (**C**) Superimposition of the 71–119 sequences of N-acyltransferase superfamily structure within Eis_*Mtb* (colored in red) onto the structure within aaNAT2 (colored in yellow). (**D**) Superimposition of the N-acyltransferase superfamily structure within Eis_*Msm* (colored in red) onto the structure within aaNAT2 (colored in yellow). (**E**) Superimposition of the 71–119 sequences of the N-acyltransferase superfamily structure within Eis_*Mtb* (colored in red) and the structure within Eis_*Msm* (colored in blue) onto the structure within aaNAT2 (colored in yellow).

The sequence information of N-acyltransferase superfamily region from National Center of Biotechnology Information and Protein Data Bank were shown in Table 4. Despite multiple sequence alignment of N-acyltransferase superfamily region showing low pairwise sequence identity (Fig. 3A), superimposition of the structures of N-acyltransferase superfamily within Eis_*Mtb*, Eis_*Msm*, Eis_*Sen*, and aaNAT revealed that Eis enzymes was very similar to aaNATs (Fig. 3B–E). Superimposition of the N-acyltransferase superfamily structure of Eis_*Mtb* with the structure of aaNAT2 showed that r.m.s. deviation was 4.636 Å for 33 Cα (Fig. 3B). N-acyltransferase superfamily region in Eis_*Mtb* increased by 25 amino acids compared with that of aaNAT2, so new superimposition of the 71–119 sequences of the N-acyltransferase superfamily structure in *Eis*_*Mtb* with the structure in aaNAT2 showed that r.m.s. deviation was 3.724 Å for 43 Cα (Fig. 3C) after deleting redundant amino acids. Superimposition of the N-acyltransferase superfamily structure of Eis_*Msm* with the structures of aaNAT2 showed that r.m.s. deviation was 2.355 Å for 46 Cα (Fig. 3D). The consequences of superimposition analysis illustrated that N-acyltransferase superfamily structure in Eis enzymes is very similar to the structure of aaNAT2 especially *Eis*_*Msm* is the most similar because of their the lowest r.m.s. deviation value, which further indicated that Eis enzymes are capable of acetylating arylalkylamines as well as aaNATs.

**Table 4:** Information of aaNAT2 from *Ae. aegypti* and Eis proteins from *M. tuberculosis, M. smegmatis and S. erythraea*.

Protein	Source	PDB.ID	Region	Sequence
aaNAT2	*Aedes aegypti*	4FD6	N-Acyltransferase (119–167)	Inlfkqfdvdkfeirilsvdsrfrgkglakkliekseelaldrgfqvm
Eis_*Mtb*	*Mycobacterium tuberculosis*	3R1K	N-Acyltransferase (46–119)	Vvvrdgagpgsevvgmalymdlrltvpgevvlptaglsfvavapthrrrgllramcaelhrriadsgypvaalh
Eis_*Msm*	*Mycobacterium smegmatis*	4QB9	N-Acyltransferase (71–119)	Vpggevlpvagisfvavapthrrrgvlramytelhdriaragyplavlt
Eis_*Sen*	*Saccharopolyspora erythraea*		N-Acyltransferase (48–113)	Iaafdqevpvggvslyprvltvpgalvpvagvasvgvapthrrrgiltammrrqladlheqgrepv

Our study revealed that Eis enzyme was a novel family of arylalkylamine N-acetyltransferase (aaNAT, EC 2.3.1.87) catalyzing the generation of N-acylarylalkylamides from the short chain acyl-CoA and a broad array of corresponding arylalkylamine substrates, although the catalytic efficiency was lower than that of the typical aaNATs. As well as aaNAT2, Eis was less discriminatory for the amine substrates including dopamine, histamine, tyramine, octopamine, phenethylamine, tryptamine, and serotonin. Actually, Eis was more discriminative for the acyl-CoA substrates, only showing activity to acetyl-CoA and propionyl-CoA and the k_cat_/K_m_ values for acetyl-CoA is ≥3-fold higher than that of propionyl-CoA. Eis enzymes showed an ordered sequential mechanism, similar to the catalytic mechanism of typical aaNAT^15,16^. However an arylamine N-Acetyltransferase from *Mycobacterium tuberculosis* revealed the bi-bi ping-pong kinetic mechanism^18^. In addition, the evolution analysis and structural comparison indicated that Eis is capable of acetylating arylalkylamines, as a novel family of arylalkylamine N-acetyltransferase. Considering arylalkylamines such as histamine plays a fundamental role in immune reactions^19^, future work linking of AANAT activity of Eis proteins to their physiological function will broaden our understanding of gram-positive pathogen-host interactions. These findings shed insights into the molecular mechanism of Eis and reveal potential clinical implications for many gram-positive pathogens.

## Materials and Methods

### Bacterial strains and reagents

*S. erythraea* NRRL2338 (from DSM 40517), *M. smegmatis* MC2 155, and *M. avium* subsp were used in this study. Strains were grown in the minimal medium (Evans) containing 25 mM TES, 2 mM citric acid, 10 mM KCl, 0.25 mM CaCl_2_, 1.25 mM MgCl_2_, 2 mM Na_2_SO_4_, 1 mM Na_2_MoO_4_, 0.5% trace elements, 2.5% (m/v) glucose, 2 mM NaH_2_PO_4_, and 10 mM NaNO_3_ (pH 7.2). The pET28a plasmid was purchased from Novagen (Gibbstown, NJ). Chemical reagents, CoA, acetyl-CoA, propionyl-CoA, butyryl-CoA, octanoyl-CoA, oleoyl-CoA, tyrosol, 5,5′-dithio-bis-(2-nitrobenzoic acid) (DTNB), dopamine, tyramine, octopamine, phenethylamine, serotonin, histamine, N-acetylhistamine, kanamycin and isopropyl-β-d-thiogalactopyranoside (IPTG) were purchased from Sigma Aldrich (St. Louis, MO, USA). Restriction enzymes including HindIII and EcoRI, T4 DNA ligase, and Phusion DNA polymerase, were purchased from TaKaRa Biotechnology Co., Ltd. (Dalian, China). The chemically competent *E. coli* DH5α and BL21 (DE3) strains were purchased from TransGen Biotech (Beijing, China). Nickel-ni-trilotriacetic acid columns were purchased from Merck (Darmstadt, Germany) and the amicon Ultra-430K cutoff centrifugal devices were purchased from Millipore Corp. (Billerica, MA, USA). UV-Vis absorption was measured by a microplate reader Synergy^TM^ Mx (Bio-Tek Instruments, Winooski, VT) using a clear flat bottom 96-well microplate (Greiner, Germany).

### Cloning, overexpression, and purification of Eis proteins

The *eis* gene of *Mtb* and the *aaNAT2* gene of *Aedes aegypti* was synthesized by Shanghai Sangon Biotechnology Co. Ltd. (Shanghai, China). The gene encoding Eis_*Sen* and Eis_*Msm* were amplified from genomic of *S. erythraea* and *M. smegmatis* by polymerase chain reaction (PCR) respectively. *E. coli* BL21 harboring different genes was grown at 37 °C in LB broth with 50 μg/mL kanamycin. When the optical density (OD_600_) of culture reached 0.6, IPTG (0.5 mM) was added to induce protein expression and then cells were grown for an additional 16 h at 20 °C. Bacterial cells were harvested by centrifugation at 5000 rpm for 20 min, and the cell pellet was rinsed thrice before sonication using phosphate buffered saline (PBS) buffer (137 mM NaCl, 2.7 mM KCl, 10 mM Na_2_HPO_4_, 1.8 mM KH_2_PO_4_ at pH 8.0). His-binding affinity chromatography resin columns were used for purification, with the resin first washed with 20 mM Tris–HCl, then 20 mM Tris–HCl containing 20 mM imidazole at pH 8.0, and finally being eluted with 250 mM imidazole in Tris–HCl (pH 8.0). The purified enzymes were obtained by filtered through a nickel-ni-trilotriacetic acid column. The protein concentration was monitored by the BCA method.

### Kinetic mechanism and inhibitor analysis

The enzyme activity was determined based on the detection of CoASH generated during acetyl transfer by reaction with the thiol reagent DTNB^16^. This assay was performed in the PBS buffer (pH 7.0) with 0.5 mM acetyl-CoA, 2 mM DTNB, and variable arylalkylamines (0.01–20 mM) at 25 °C. A parallel assay was performed in the PBS buffer (pH 7.0) with 5 mM arylalkylamine hold the highest affinity, 2 mM DTNB, and variable Acyl-CoAs (0.01–800 μM) at 25 °C. Reactions were initiated with enzyme (0.25 μM) that had been prediluted (10–100-fold) with PBS and maintained on ice during the assay. Assays were measured using a UV-vis microplate reader in a 96-well plate. In the real-time monitoring of the enzymatic reaction, measurements at 412 nm were acquired every 10 s for 300 s. The data were fitted to an enzyme kinetics Michaelis-Menten curve to determine Km and V_max_ values using GraphPad Prism 5 (GraphPad Software, Inc., La Jolla, CA). Dead-end inhibitor analysis was conducted for both oleoyl-CoA and tyrosol. The inhibition patterns were determined by holding one substrate (acetyl-CoA or histamine) at a fixed concentration and varying the concentration of the other substrate, with each set conducted at a different fixed concentration of the inhibitor^14^. The data were fitted to an enzyme kinetics inhibition curve to determine Ki values using GraphPad Prism 5.

### Evolution analysis and structural comparison of the N-acyltransferase superfamily

The phylogenetic tree was generated using the Molecular Evolutionary Genetics Analysis. The gi number from NCBI (The National Center for Biotechnology Information) database (https://www.ncbi.nlm.nih.gov/) is provided for each protein sequence. The protein crystal structures were downloaded from Protein Data Bank (http://www.rcsb.org/pdb/home/home.do) and the information of N-acyltransferase superfamily region were got from NCBI. The multiple sequence alignment of N-acyltransferase superfamily region was obtained by BioEdit (Isis Pharmaceuticals, Inc.). Superposition of structures was done using Discovery Studio 4.0 Client (Accelrys Software Inc., San Diego, CA) and the r.m.s. deviation were generated using UCSF Chimera (http://www.cgl.ucsf.edu/chimera/).
